# Effect of Pond-Based Rice Floating Bed on the Microbial Community Structure and Quality of Water in Pond of Mandarin Fish Fed Using Artificial Diet

**DOI:** 10.3390/biology13070549

**Published:** 2024-07-21

**Authors:** Lijin Jiang, Mengmeng Yi, Zhiyong Jiang, Yuli Wu, Jianmeng Cao, Zhigang Liu, Zhang Wang, Maixin Lu, Xiaoli Ke, Miao Wang

**Affiliations:** 1 Key Laboratory of Tropical & Subtropical Fishery Resource Application & Cultivation, Ministry of Agriculture and Rural Affairs, Guangdong Provincial Key Laboratory of Aquatic Animal Immunology and Sustainable Aquaculture, Pearl River Fisheries Research Institute, Chinese Academy of Fishery Science, Guangzhou 510380, China; jiang19990311@163.com (L.J.); ymmmb@163.com (M.Y.); caojianmeng@aliyun.com (J.C.); wenliugang@163.com (Z.L.); wangzh19930124@163.com (Z.W.); mx-lu@163.com (M.L.); xiaolike2012@163.com (X.K.); 2 Guangdong Agricultural Technology Extension Center, Guangzhou 510520, China; zyjiang0101@163.com (Z.J.); xiaowugongzuo@163.com (Y.W.)

**Keywords:** pond-based rice floating bed, mandarin fish, artificial diet, water quality, microbial community structure

## Abstract

**Simple Summary:**

Simple Summary: This study presents the initial findings on how rice cultivation impacts water quality and the microbial community structure in ponds where mandarin fish are being fed an artificial diet. The results indicated that rice cultivation plays a significant role in reducing nutrient salt levels in the water, thus regulating the overall aquatic environment. Furthermore, the cultivation of rice has been found to alter the composition of microbial communities in the water, leading to a decrease in diversity and affecting the abundance of bacteria involved in carbon, nitrogen, and phosphorus metabolism. The study identified Proteobacteria, Actinobacteria, Firmicutes, Bacteroidetes, and Deinococcus-Thermus as the dominant bacterial phyla in ponds where mandarin fish are being fed an artificial diet. Additionally, it was observed that Proteobacteria showed a particular preference for proliferation in the aquatic environment with rice cultivation on the water surface. These results provide a valuable foundation for the potential application of aquaculture practices involving mandarin fish fed an artificial diet and rice floating beds.

**Abstract:**

The culture of mandarin fish using artificial feed has been gaining increasing attention in China. Ensuring good water quality in the ponds is crucial for successful aquaculture. Recently, the trial of pond-based rice floating beds (PRFBs) in aquaculture ponds has shown promising results. This research assessed the impact of PRFBs on the microbial community structure and overall quality of the aquaculture pond, thereby enhancing our understanding of its functions. The results revealed that the PRFB group exhibited lower levels of NH_4_^+^-N, NO_2_^−^-N, NO_3_^−^-N, TN, TP, and Alk in pond water compared to the control group. The microbial diversity indices in the PRFB group showed a declining trend, while these indices were increasing in the control group. At the phylum level, there was a considerable increase in Proteobacteria abundance in the PRFB group throughout the culture period, suggesting that PRFBs may promote the proliferation of Proteobacteria. In the PRFB group, there was a remarkable decrease in bacterial populations related to carbon, nitrogen, and phosphorus metabolism, including genera *Rhodobacter*, *Rhizorhapis*, *Dinghuibacter*, *Candidatus* Aquiluna, and *Chryseomicrobium* as well as the CL500_29_marine_group. Overall, the research findings will provide a basis for the application of aquaculture of mandarin fish fed an artificial diet and rice floating beds.

## 1. Introduction

The mandarin fish or Chinese perch, scientifically known as *Siniperca chuatsi* (Basilewsky, 1855) (Perciformes: Percichthyidae), is a carnivorous species. Traditionally, the cultivation of mandarin fish relied on the use of live bait fish. However, the inadequate supply of bait fish has significantly hindered the growth of the mandarin fish aquaculture industry. It is estimated that four acres of bait fish ponds are required for every acre of mandarin fish cultivation. The feeding ratio for mandarin fish is high, with approximately four to five pounds of bait fish needed to produce one pound of mandarin fish. Consequently, this has resulted in increased costs for farmers. Fortunately, recent advancements in artificial feed technology specifically designed for mandarin fish have shown promising improvements in addressing these challenges. By 2022, aquaculture of mandarin fish using artificial feed in China has reached a rate of approximately 5%, yielding an output of 20,000 tons, according to the China Society of Fisheries [1]. Research has indicated that the nutritional composition of mandarin fish reared with artificial feed, particularly protein and amino acid content, was comparable to those fed bait fish diet. Additionally, these fish demonstrated a superior fatty acid profile with elevated levels of eicosapentaenoic acid (EPA) and docosahexaenoic acid (DHA) reported by Niu et al. (2023) [2]. However, it has been observed by Li et al. (2017) [3] that the diversity of intestinal microorganisms was lower in mandarin fish fed artificial feeds compared to those fed bait fish. Mandarin fish following an artificial diet displayed reduced gut microbial diversity and lower levels of certain enzymes, such as alkaline phosphatase (AKP), catalase (CAT), and lysozyme (LZM). Despite this progress, further research is urgently needed to optimize artificial feed-based aquaculture methods for mandarin fish.

The culture of mandarin fish demands high water quality standards. A study conducted by Gao et al. (2000) [4] indicated that mandarin fish ponds should maintain a suspended solids concentration of no more than 25 mg/L, a pH level between 6.5 and 8.5, and a dissolved oxygen level exceed 5 mg/L. Mandarin fish exhibited a high sensitivity to un-ionized ammonia, requiring concentrations below 0.025 mg/L. Hydrogen sulfide, NH_4_^+^-N, and NO_2_^−^-N levels should not exceed 0.1 mg/L, 1.0 mg/L, and 0.1 mg/L, respectively [5]. Thus, improvements in the water environmental conditions of mandarin fish aquaculture ponds are vital for the sustainable development of the industry.

Extensive studies have reported on the application of plant floating beds on environmental restoration. They showed that the floating bed cultivation of aquatic plants could reduce the levels of N, P, and other contaminants in water bodies [6] and was effective in treating pond water [7], eutrophic water [8], industrial wastewater [9], agricultural wastewater [10], and domestic sewage [11]. In this study, we utilized the pond-based rice floating beds (PRFBs) system for the cultivation of mandarin fish using artificial feed. This method differs from the traditional integrated rice-fish farming (IRFF) technique, which involves raising fish in rice paddy fields. IRFF has been successful in the production of various fish species, including Nile tilapia (*Oreochromis niloticus*) (Linnaeus, 1758) [12], African catfish (*Clarias gariepinus*) (Burchell, 1822) [13], yellow catfish (*Pelteobagrus fulvidraco*) (Richardson, 1846) [14], common carp (*Cyprinus carpio*) (Linnaeus, 1758) [12], mrigal carp (*Cirrhinus mrigala*) (Hamilton, 1822) [15], Asian seabass (*Lates calcarifer*) (Bloch, 1790) [16], major South Asian carp (*Catla catla*) (Hamilton, 1822) [15], rohu (*Labeo rohita*) (Hamilton, 1822) [15], and Dojo loach (*Misgurnus anguillicaudatus*) (Cantor, 1842) [17]. Several studies have examined the effects of PRFBs on the quality of pond water. For example, Goda et al. (2023) [18] discovered that PRFBs significantly enhanced the utilization of nitrogen (N) and phosphorus (P) in the water, while Zhu et al. (2023) [19] emphasized that this method effectively decreased the levels of N and P in water bodies. Although these studies suggested the potential of PRFBs to improve water quality, there has been a lack of comprehensive research on its impact on water quality and the associated microbial communities. Therefore, the objective of this study was to investigate the effects of PRFBs on water quality and the structure of microbial communities in mandarin fish ponds. This research provides insights for the utilization of PRFBs in aquaculture practices aimed at cultivating mandarin fish using an artificial diet.

## 2. Materials and Methods

### 2.1. Experimental Design

**Figure 1:**
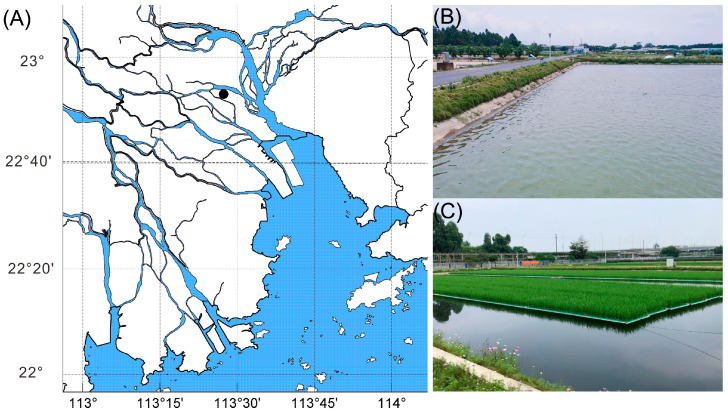
(**A**) The sampling location; (**B**) the control pond; (**C**) the PRFB pond. The black dot indicates the sampling location.

The aquaculture experiment was carried out at a farm located in Panyu, Guangzhou, Guangdong Province, China (Figure 1). Mandarin fish with an average body weight of 12.20 ± 8.43 g were fed an artificial feed in six ponds. Each pond was designated with a label: control-A, control-B, control-C, PRFB-A, PRFB-B, and PRFB-C. Notably, PRFB-A, PRFB-B, and PRFB-C had a rice cultivation area that accounted for 30% of the total pond area, with rice variety Meixiangzhan No. 2 being grown on high-density polyethylene floating beds (Table 1). All ponds sourced their aquaculture water from a reservoir. On 30 July 2022, measurements were taken to assess water quality, including total phosphorus (TP), alkalinity (Alk), total nitrogen (TN), nitrite–nitrogen (NO_2_^−^-N), nitrate–nitrogen (NO_3-_-N), and total ammonia nitrogen (NH_4_^+^-N), and water from the reservoir was introduced into the experimental ponds (Table 2). Concurrently, rice cultivation was initiated on the floating beds within the PRFB group ponds.

Fish were fed twice a day at 2% to 4% of body mass using a commercial diet purchased from Foshan Nanhai Jieda Feed Co., Ltd. (Foshan, China). (Supplemental Table S1). The feeding amount was adjusted accordingly as the growth decreased, taking into consideration factors such as weather, water quality, and the feeding status of the fish. During the experiment, the water temperature ranged from 30 to 33 °C, pH was 7.5–8.2, the dissolved oxygen (DO) concentration was 7.56–8.70 mg/L, and total suspended solids (TSS) were 29.60–38.32 mg/L.

### 2.2. Sampling

We conducted a total of nine samplings, which were divided into three sampling periods. The first, second, and third samplings took place every three days from 20 August to 26 August (period I), 20 September to 26 September (period II), and 20 October to 26 October 2022 (period III), respectively. There were 54 water samples in total. We collected equal volumes of water from 30 cm below the surface at the four corners of each pond. The collected water was thoroughly mixed and then stored in sterile 250 mL water sampling bags. These water samples were later analyzed for their quality and filtered for sequencing. We utilized a 0.22 µm filter membrane with a filtration volume of 200 mL. The filter membranes were preserved at −80 ºC in freezers for subsequent high-throughput sequencing using the Illumina platform.

### 2.3. Method for Water Quality Measurement

We utilized methyl red as an indicator and conducted a titration on a 100 mL water sample using a known concentration of diluted hydrochloric acid to determine the alkalinity of the water. The concentrations of NH_4_^+^-N, NO_2_^−^-N, NO_3_^−^-N, and TP were measured using a multi-parameter water quality analyzer 5B-3B (Beijing Lianhua YongXing Science and Technology Development Co., Ltd., Beijing, China). The total nitrogen (TN) content was quantified using a total nitrogen analyzer LH-3B (Beijing Lianhua YongXing Science and Technology Development Co., Ltd., Beijing, China) (Table 3).

### 2.4. Illumina High-Throughput Sequencing

DNA from samples was isolated using the Quick-DNA™ Universal Kits (Zymo Research, Irvine, CA, USA). The V4 region of the 16S rRNA gene was amplified using the polymerase chain reaction (PCR) with the 515f/806r primer set: 5′-GTGCCAGCMGCCGCGGTAA-3′ and 5′-GGACTACHVGGGTWTCTAAT-3′. The library was constructed using the TruSeq^®^ DNA PCR-Free Sample Preparation Kit (Illumina, Inc., San Diego, CA, USA) and quantified using Qubit and Q-PCR. Sequencing was performed on a NovaSeq 6000 (Illumina, San Diego, CA, USA) platform.

### 2.5. Data Processing and Statistical Analysis

High-throughput sequencing data were subjected to quality control (QC) using the Illumina standard workflow to ensure the integrity of the raw data. The filtered reads were then assembled into tags. Subsequently, the tags underwent further filtration, resulting in a set of clean tags for subsequent analysis.

The Usearch software (version 10.0) was utilized to cluster tags, operating at a 97% similarity threshold, to generate operational taxonomic units (OTUs). The taxonomic labels were assigned to these OTUs by annotating them using the Silva (Release132) taxonomic database (http://www.arb-silva.de, accessed 13 February 2023). Subsequently, the representative sequences for each OTU were aligned against microbial reference databases to obtain species classification information. The community composition of each sample was then analyzed at various taxonomic levels. To generate species abundance tables at different taxonomic ranks and calculate α diversity indices, the QIIME2 software (version 2019.4) was utilized. Based on the species abundance matrix and the classification data, the partial least squares discriminant analysis (PLS-DA) model was created by R to study the structural variation in microbial communities. LEfSe (linear discriminant analysis effect size) was performed to detect differentially abundant taxa across groups using the default parameters. Spearman’s rank correlation was performed to identify correlations between the water quality and relative abundances of genera among water microbiota. The “pheatmap” package in R was used to generate heat maps, and the “correlation” clustering method was used for the “pheatmap” function. Co-occurrence networks were inferred based on a Spearman correlation matrix and constructed using only significant correlation. The cutoff for correlation coefficients was determined to be 0.6, and the cutoff for *p*-values was 0.05. To further explore the impact of microbial community change, functional prediction with FAPROTAX annotation tool (https://pages.uoregon.edu/slouca/LoucaLab/archive/FAPROTAX/lib/php/index.php, accessed 10 July 2024) was performed. The predicted results were visualized by an LEfSe by R.

The experimental results were expressed as mean values ± standard error.

## 3. Results

### 3.1. Analysis of Water Quality Parameters

**Figure 2:**
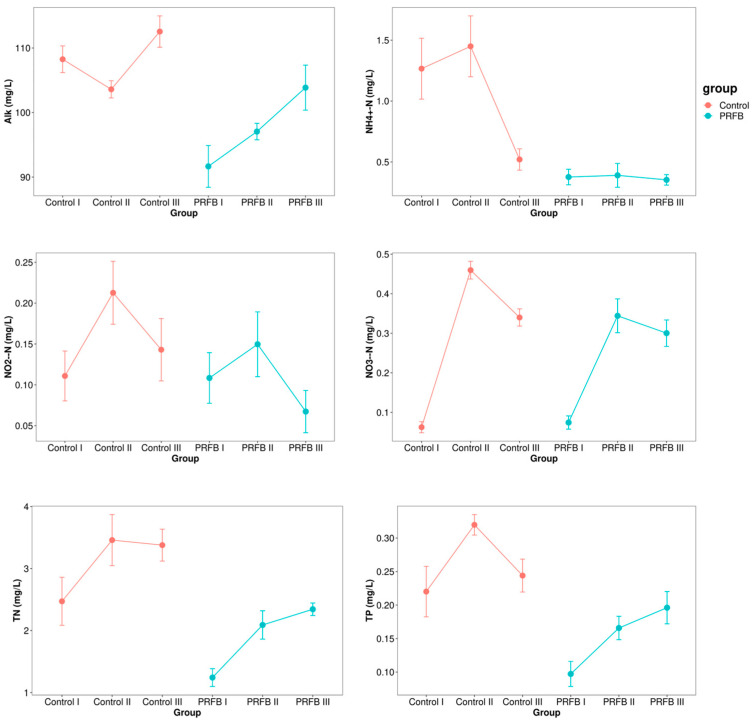
Changes in water quality in different groups. Note: Control I, Control II, and Control III represent the sample numbers in the control group for periods I, II, and III respectively. PRFB I, PRFB II, and PRFB III represent the sample numbers in the PRFB group for periods I, II, and III, respectively.

The results indicated that the alkalinity (Alk) concentration in the aquaculture water of the control group exhibited an initial decline followed by an increase, whereas it consistently increased in the PRFB group. Conversely, the NH_4_^+^-N concentration in the control group experienced an initial rise followed by a decrease, while it demonstrated negligible changes in the PRFB group. The concentrations of NO_2_^−^-N and NO_3_^−^-N in both the control and PRFB groups exhibited a similar pattern with an initial increase followed by a decrease. Nevertheless, overall, the PRFB group displayed lower levels of NO_2_^−^-N, NO_3_^−^-N, TN, and TP in comparison to the control group (Figure 2). Water quality measurements taken in individual ponds during different sampling periods are presented in the Supplemental Table S2. The results indicated that there were no significant differences between the control ponds within the same period. For the PRFB ponds, there were significant differences in ALK and NO_2_^−^-N levels during period III, with all others showing no significant differences within the same period. To further explore these findings, the levels of TN and TP in water samples from the two groups during period III were compared. It was revealed that the TN and TP levels in the PRFB ponds decreased by approximately 30.62% and 19.63%, respectively, in comparison to the control group by the end of the experiment.

### 3.2. Analysis of Illumina High-Throughput Sequencing Results

#### 3.2.1. α-Diversity Analysis

**Figure 3:**
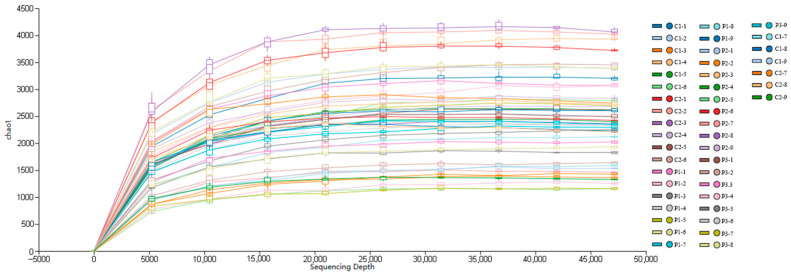
Rarefaction curve. Note: C represents the sample number in the control group. P represents the sample number in the PRFB group.

Pond control-C did not achieve successful aquaculture; hence, two control ponds were included in the analysis of Illumina high-throughput sequencing. There were six replicates for the control group and nine replicates for the PRFB group in each sampling period in total. Rarefaction curve analysis showed that the curves eventually reached a considerably flat plateau, demonstrating that the sample sizes of sequencing were sufficient and covered the full diversity of the samples (Figure 3).

**Figure 4:**
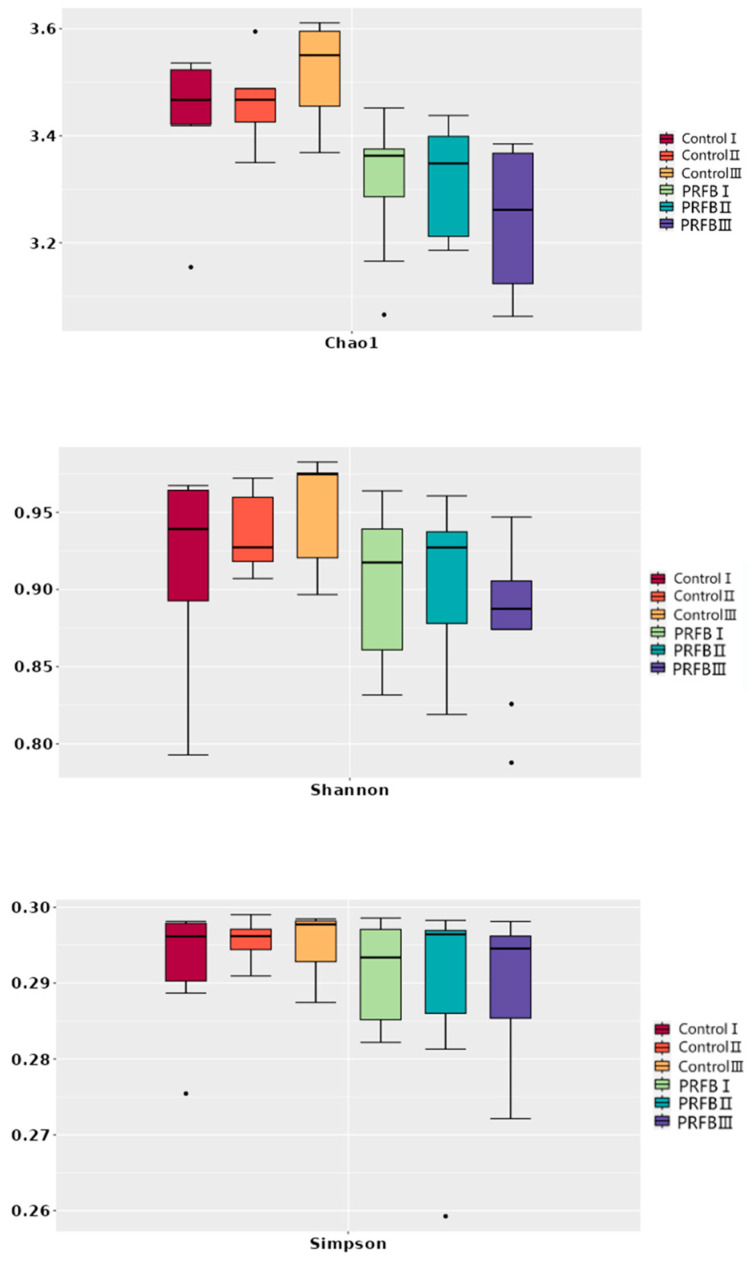
α- Diversity index analysis. Note: Control I, Control II, and Control III represent the sample numbers in the control group for periods I, II, and III respectively. PRFB I, PRFB II, and PRFB III represent the sample numbers in the PRFB group for periods I, II, and III, respectively.

The results demonstrated that in period III, the Chao1 index of the control group increased, while that of the PRFB group decreased. The Simpson index remained relatively stable in the control group during period I and II but increased in period III. The Simpson index in the PRFB group increased in period II and decreased in period III. Overall, the richness and diversity of bacterial communities of the control group were found to be higher than that of the PRFB group (Figure 4).

#### 3.2.2. β-Diversity Analysis

**Figure 5:**
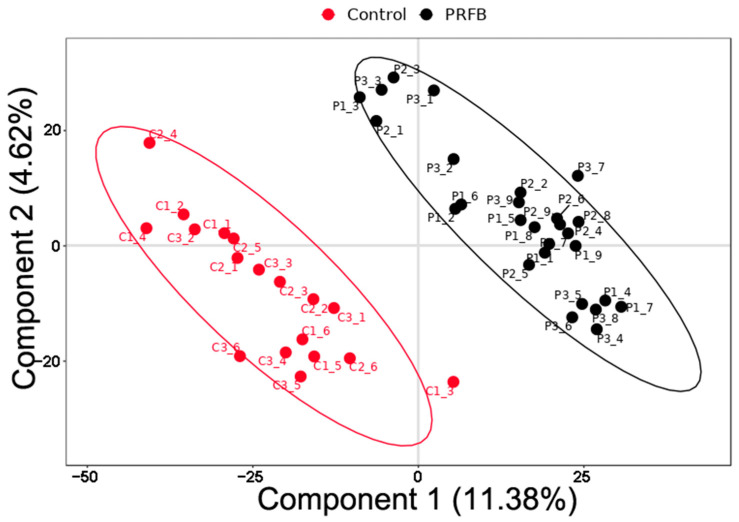
PLSDA analysis between different groups.

The microbial composition differences among the samples were analyzed using the PLSDA analysis method. The results of the PLSDA analysis for the control group and the PRFB group showed that PCA 1 accounted for 11.38% of the variations observed in the samples, while PCA 2 accounted for 4.62% of the variations. Significant differences in the structure of the microbial communities were observed between the control group and the PRFB group (Figure 5).

### 3.3. Spearman Correlation Analysis between Water Quality Parameters and Microbial Communities at the Genus Level

**Figure 6:**
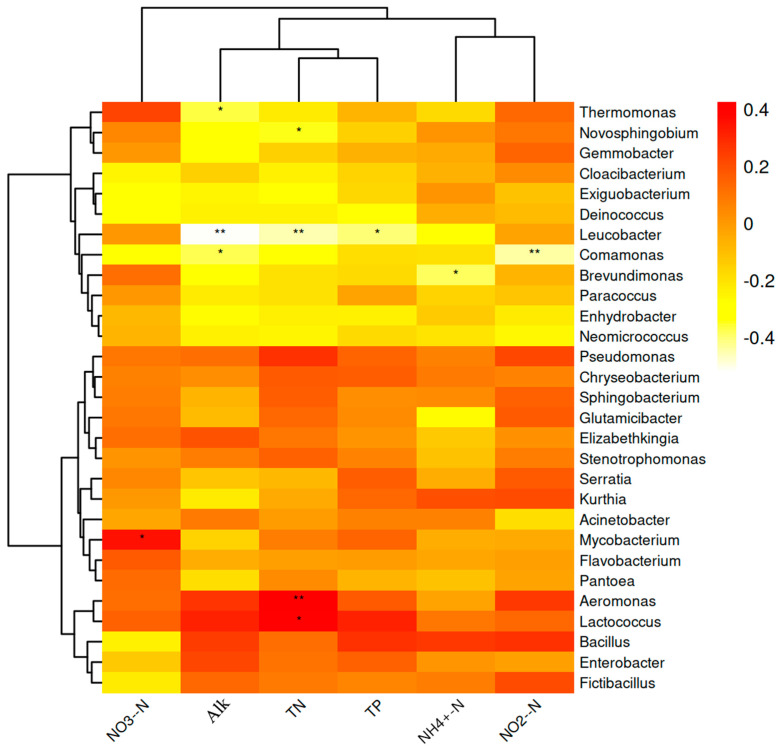
Spearman correlation analysis between water quality parameters and microbial communities at the genus level. Note: * represents *p* < 0.05, ** represents *p* < 0.01, and the correlation coefficient R > 0.3.

The Spearman correlation analysis was conducted to investigate the relationship between the abundance of the top 30 bacterial genera in the aquaculture ponds and selected water quality parameters. The results revealed a significant positive correlation between NO_3_^−^-N and *Mycobacterium* (*p* < 0.05). Moreover, Alk showed a significant negative correlation with *Thermomonas* (*p* < 0.05), *Leucobacter* (*p* < 0.01), and *Comamonas* (*p* < 0.05). TN exhibited a significant positive correlation with *Aeromonas* (*p* < 0.01) and *Lactococcus* (*p* < 0.05), while it showed a significant negative correlation with *Leucobacter* (*p* < 0.01) and *Novosphingobium* (*p* < 0.05). TP demonstrated a significant negative correlation with *Leucobacter* (*p* < 0.05). Additionally, NH_4_^+^-N exhibited a significant negative correlation with *Brevundimonas* (*p* < 0.05). NO_2_^−^-N was significantly negatively correlated with *Comamonas* (*p* < 0.01) (Figure 6).

### 3.4. Analysis of Microbial Community Composition

**Figure 7:**
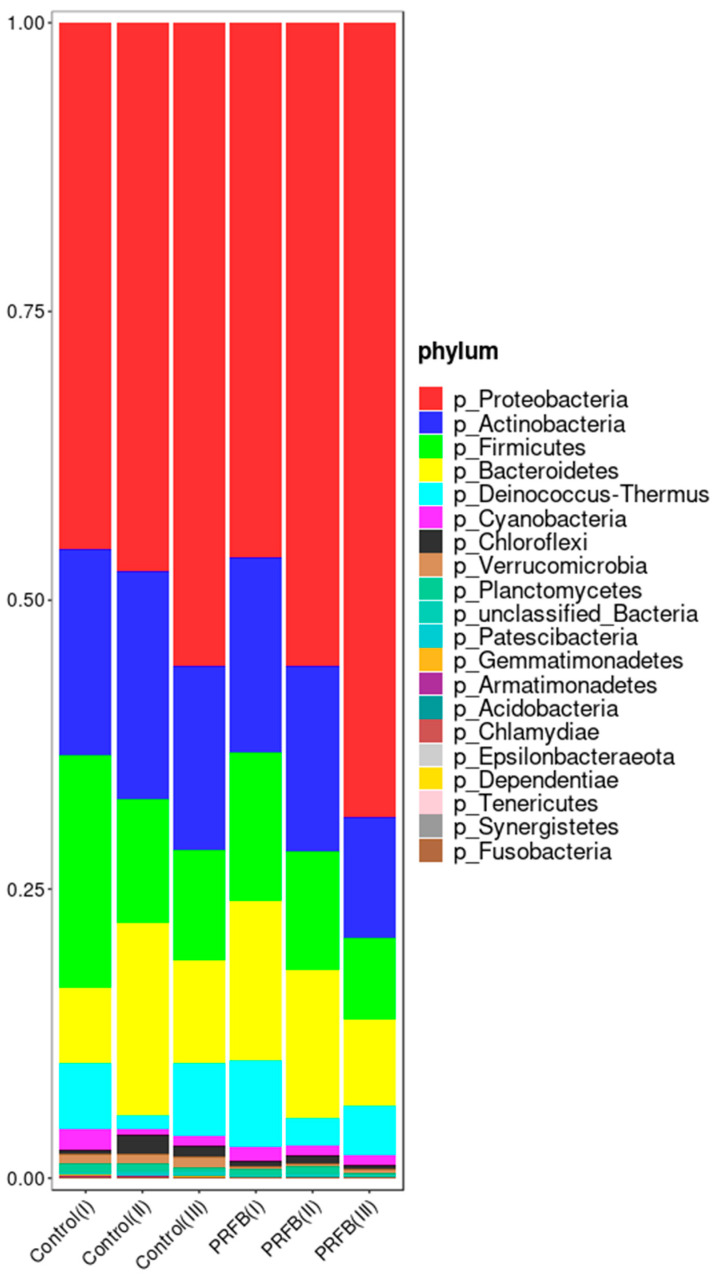
Relative abundance of microbiota at the phylum level. Note: Control I, Control II, and Control III represent the sample numbers in the control group for periods I, II, and III, respectively. PRFB I, PRFB II, and PRFB III represent the sample numbers in the PRFB group for periods I, II, and III, respectively.

The results revealed that the predominant phyla present in the pond water of both the control group and the PRFB group were similar. Proteobacteria, Actinobacteria, Firmicutes, Bacteroidetes, and Deinococcus-Thermus exhibited high relative abundances in both the control and PRFB groups. Additionally, it was observed that as the aquaculture experiment progressed, the abundance of Proteobacteria increased in both the control and PRFB groups (Figure 7).

**Figure 8:**
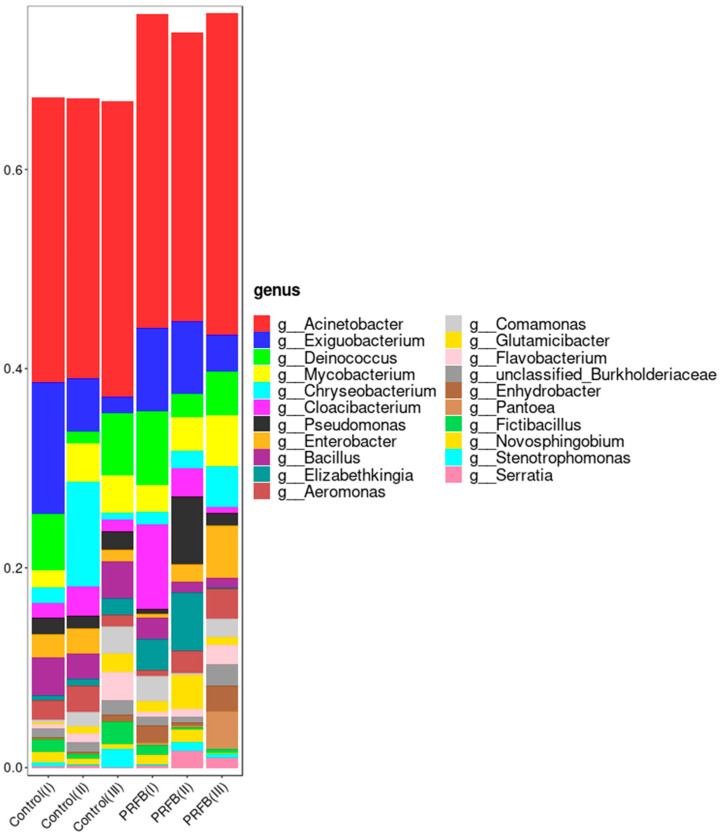
Relative abundance of microbiota at the genus level. Note: Control I, Control II, and Control III represent the sample numbers in the control group for periods I, II, and III, respectively. PRFB I, PRFB II, and PRFB III represent the sample numbers in the PRFB group for periods I, II, and III, respectively.

On the genus level, it was observed that during the aquaculture experiment, *Acinetobacter* was a dominant genus and remained stable in both the control and PRFB groups with no significant changes in abundance during periods I, II, and III. *Exiguobacterium* showed a noticeable decrease in abundance as the experiment progressed in both the control and PRFB groups. The abundance of *Deinococcus* showed a pattern of decrease followed by an increase in both the control and PRFB groups. The abundance of *Mycobacterium* exhibited an increasing trend in both the control and PRFB groups. In the control group, the abundance of *Chryseobacterium* initially increased and then decreased. In the PRFB group, the abundance of *Chryseobacterium* continued to increase. The abundance of *Cloacibacterium* increased and then decreased in the control group, while it continuously decreased in the PRFB group. In the control group, the abundance of *Pseudomonas* initially decreased and then increased. However, in the PRFB group, the abundance of *Pseudomonas* initially increased and then decreased. In the control group, the abundance of *Enterobacter* showed a decreasing trend, while in the PRFB group, it showed an increasing trend (Figure 8).

### 3.5. Analysis of Differential Bacterial Genera

**Figure 9:**
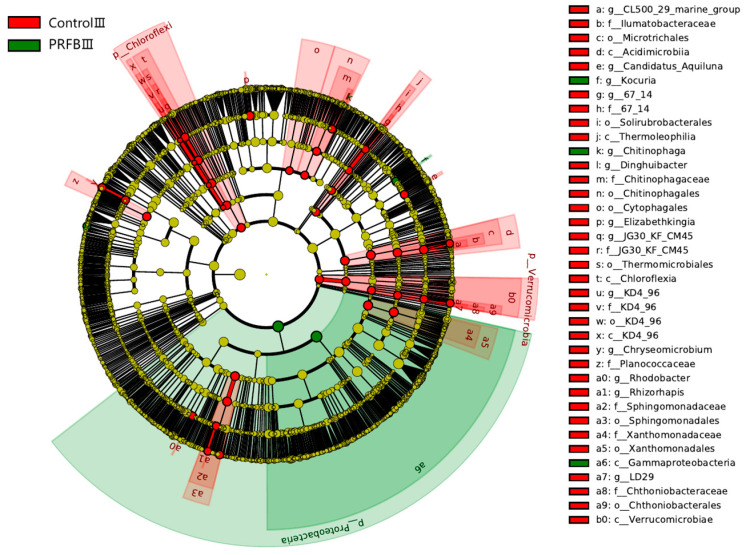
LefSe analysis of microbial communities. Note: Control III and PRFB III represent the sample numbers for the control group and PRFB group, respectively, during the third sampling period; LDA discriminant analysis score threshold is 3, *p* < 0.05.

LEfSe applied linear discriminant analysis to identify taxa with significant differences in abundance. The results showed that there were significant differences in the relative abundance of 3 phyla and 13 genera between the control III and PRFB III samples (*p* < 0.05). Among them, the relative abundances of Chloroflexi and Verrucomicrobia in the control III sample were significantly higher than those in the PRFB III sample, and the relative abundance of Proteobacteria was significantly lower than that in the PRFB III sample (*p* < 0.05). In addition, the relative abundances of *Rhodobacter*, KD4-96 (Chloroflexi), 67-14 (Actinobacteria), *Rhizorhapis*, *Dinghuibacter*, *Candidatus*_Aquiluna (Actinobacteria), LD29 (Verrucomicrobia), *Chryseomicrobium*, JG30_KF_CM45 (Chloroflexi), CL500-29_marine_group (Actinobacteria), and *Elizabethkingia* were significantly higher in the control III sample compared to the PRFB III sample. On the other hand, *Kocuria* and *Chitinophaga* showed significantly higher relative abundances in the PRFB III sample (*p* < 0.05) (Figure 9).

### 3.6. The Correlation Network Analysis

**Figure 10:**
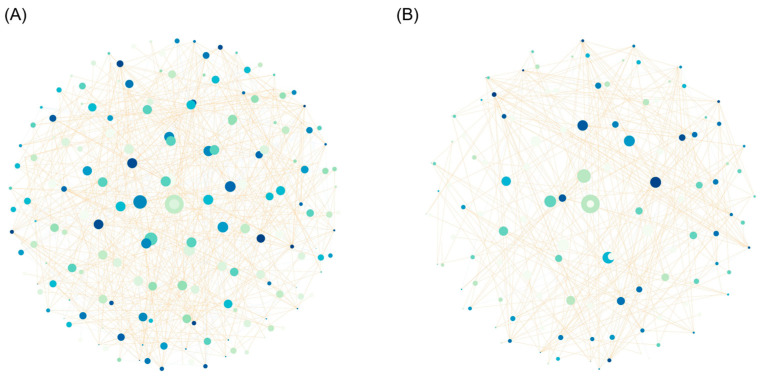
Correlation network analysis of water microbiota based on genus level: (**A**) control group (nodes  =  181, edges  =  507) and (**B**) PRFB group (nodes  =  124, edge  =  277). Spearman’s R  >  0.6; *p*  <  0.05.

Control samples had more nodes (181) and edges (506) as well as higher connectance (0.0257) compared to the PRFB samples (nodes  = 124, edges  =  277, and connectance  =  0.0136) (Figure 10). These results indicated that microorganisms from control groups are more interrelated than those from PRFB groups.

### 3.7. Function Analysis

**Figure 11:**
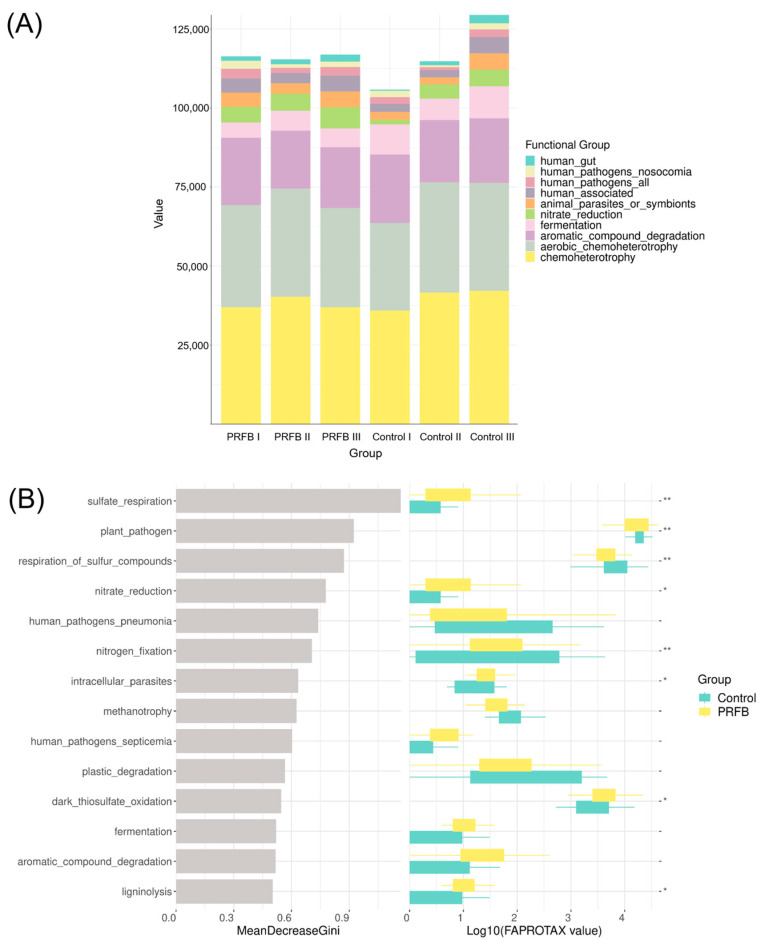
FAPROTAX analysis of the water microbiota. (**A**) The relative abundance of top 10 functions; (**B**) LefSe analysis of functional composition of the water microbiota based on FAPROTAX. * *p* < 0.05; ** *p* < 0.01.

For FAPROTAX analysis, chemoheterotrophy, aerobic chemoheterotrophy, aromatic compound degradation, fermentation, and nitrate reduction were found among the top 5 most abundant functions (Figure 11A). The results of FAPROTAX showed significant changes in the abundance of some metabolic pathways of microbiota community of the water samples (Figure 11B). Most of these pathways involved nitrogen and sulfur metabolism, including sulfate respiration, the respiration of sulfur compounds, nitrate reduction, and nitrogen fixation. Those genes increased in the PRFB group compared to the control group.

## 4. Discussion

The stability of the pond’s ecological environment is crucial for the optimal growth of fish. The physicochemical properties and microbial community structure of the aquaculture water play a vital role in ensuring the success of the aquaculture process. It is of importance to implement new culture systems to improve water resource management practices [20]. These innovative methods effectively enhance water use efficiency and nitrogen and phosphorus use efficiency, and they also facilitate adjustments to the microbial community structure [21].

The PRFB is a new integrated aquaculture system. Our study revealed that the PRFB aquaculture system demonstrated lower concentrations of Alk, NH_4_^+^-N, NO_2_^−^-N, NO_3_^−^-N, TN, and TP compared to the control group. These findings suggest that rice cultivation in the PRFB system effectively absorbs nutrients from the water, and it enhances environmental nutrient utilization efficiency. These conclusions are in line with previous studies on IRFF and floating beds of aquatic plants [7,9,22,23,24]. Rice is capable of absorbing and utilizing NH_4_^+^-N and NO_3_^−^-N through its root system [25,26]. However, there is still limited research on the interaction mechanism between Alk and rice in water. Some studies have suggested that rice roots secrete organic acids [27]. Furthermore, the conversion of NH_4_^+^-N and NO_2_^−^-N in water is dependent on the consumption of Alk, including processes such as nitrification, denitrification, and algal photosynthesis [28,29]. Therefore, it can be inferred that the decrease in Alk following rice planting may be attributed to the above mechanism.

Using Spearman correlation analysis, we identified eight bacterial genera that are significantly correlated with water quality indicators and may contribute to nitrogen and phosphorus cycling. These genera included *Mycobacterium*, *Thermomonas*, *Leucobacter*, *Comamonas*, *Aeromonas*, *Lactococcus*, *Novosphingobium*, and *Brevundimonas*. Previous research has shown that certain species of *Mycobacterium*, such as *M. vaccae* and *M. smegmatis*, contained genes encoding for nitrate reductase, allowing them to convert NO_3_^−^-N to NO_2_^−^-N [30]. *Aeromonas* and *Lactococcus* carried genes that enable the stable removal of NH_4_^+^-N and TN [31]. *Novosphingobium* was an important denitrifying bacterium that was significantly positively correlated with N_2_O emissions [32]. *Brevundimonas* was effective in converting NH_4_^+^-N to NO_2_^−^-N [33]. A study conducted by Zhu et al. (2023) [34] on wastewater treatment found a negative correlation between *Leucobacter* and TP, which was consistent with the findings of our current study. Alk refers to the combined amount of acidic and alkaline substances in water, which is used to indicate the acidity or alkalinity of the water. In a sulfur-oxidizing denitrification filter, *Thermomonas* had a lower relative abundance in the Alk-raised group compared to the unadjusted group [35]. Furthermore, *Comamonas* efficiently removed NO_2_^−^-N from water [36], and Zheng et al. (2014) [37] discovered that when using *Comamonas* for the treatment of highly alkaline wastewater from a paper mill (pH 13.2~13.5), this bacterium did not require additional carbon sources for water treatment. During the treatment process, it can produce various acidic compounds, which may lead to a decrease in the alkalinity of the water.

This study presented the first application of PRFBs in mandarin fish farming. The β-diversity analysis of the microbial community revealed significant differences between the PRFB group and the control group in terms of microbial community structure. Interestingly, the study found that the PRFB group had lower α-diversity, as indicated by the Chao1, Simpson, and Shannon indices, compared to the control group. This suggested that growing rice on the pond surface in the PRFB group leads to a reduction in microbial diversity in the water. Previous studies have also reported a decrease in soil microbial diversity with IRFF [38,39,40]. Similarly, in an IRFF system in Sleman, Yogyakarta, Indonesia, it was observed that microbial diversity in the culture water decreased after rice cultivation [39]. This can be attributed to the direct absorption and utilization of nutrients by rice plants when grown on the pond surface, which could limit the growth of certain microorganisms.

The dominant bacterial phyla in both the PRFB group and control group at the end of the experiment (Period III) were Proteobacteria, Actinobacteria, Firmicutes, Bacteroidetes, and Deinococcus-Thermus, which were commonly found in water environments and sediment [41]. However, the LEfSe analysis revealed significant differences between the PRFB group and the control group in terms of the phyla Proteobacteria. Specifically, the relative abundance of Proteobacteria increased significantly in the water of the PRFB group, which was consistent with previous studies on IRFF [38,42]. Proteobacteria are known for their role in organic matter decomposition, producing various types of glycoside hydrolases and participating in nitrogen fixation to promote plant growth [43].

We have identified 13 genera that displayed significant differences in abundance based on LEfSe differential analysis. Our study revealed that the abundance of *Kocuria* and *Chitinophaga* in the PRFBs water was significantly higher compared to the control. Conversely, the abundance of *Rhodobacter*, KD4-96, 67-14, *Rhizorhapis*, *Dinghuibacter*, *Candidatus*_Aquiluna, LD29, *Chryseomicrobium*, JG30_KF_CM45, CL500_29_marine_group, and *Elizabethkingia* in the PRFBs water was significantly lower compared to the control water. *Chitinophaga* bacteria were capable of degrading chitin and converting nitrate into nitrite and nitrogen gas [44]. *Rhodobacter* bacteria belonged to the group of purple nonsulfur bacteria, many of which were capable of photosynthesis under sunlight. They typically used organic compounds as electron donors and simultaneously reduced the organic content of water during photosynthesis [45,46,47]. *Rhizorhapis* was capable of converting nitrogen gas into ammonia [48,49,50]. *Dinghuibacter* was commonly found in freshwater lakes, rivers, ponds, and other water bodies [51]. Its main sources of carbon were oligosaccharides and glucose, while ammonia and nitrate served as its nitrogen sources. Some strains of *Dinghuibacter* can also degrade toxic substances, making them potentially valuable in environmental remediation [52]. The 67–14, *Candidatus*_Aquiluna, and CL500-29_marine_groups were classified under the phylum Actinobacteria. 67–14 was an efficient degrader of various organic and inorganic carbon compounds, and it showed a significant positive correlation with phosphorus [53,54]. This suggested that 67–14 was capable of removing both organic and inorganic carbon compounds as well as phosphorus from water. Li et al. (2020) [55] discovered a positive correlation between *Candidatus*_Aquiluna and ammonia levels. The CL500-29_marine_group (Actinobacteria) was a dominant bacterial genus found in both water samples and sediments. It played a crucial role in carbon metabolism in aquatic environments by utilizing dissolved organic carbon under aerobic conditions [41,56]. Additionally, the unclassified genus KD4-96, belonging to Chloroflexi, demonstrated diverse metabolic activities and ecological functions. It was capable of biodegrading halosulfuron methyl and oxidizing nitrite [57,58]. JG30_KF_CM45 (Chloroflexi) was positively correlated with nitrate and total nitrogen [59,60,61]. LD29 (Verrucomicrobia) has been found to have a wide distribution and strong correlation with other bacterial species in a tropical estuarine reservoir, as highlighted by Xu et al. (2018) [62] and Zwart et al. (1998) [63]. In a study conducted by Mohapatra et al. (2019) [64], it was identified that LD29 primarily obtained its carbon source from *Synechococcus* in aquatic environments. The researchers speculated that LD29 may play a crucial role in the carbon cycle by decomposing organic compounds derived from phytoplankton. *Chryseomicrobium* bacteria were well known for their distinct physiological characteristics, including high tolerance to temperature and salinity, which allowed them to thrive in diverse environments [65]. Additionally, Wu et al. [66] discovered that *Chryseomicrobium* bacteria had the ability to degrade organic substances and exhibit denitrification functions. In the present research, reductions in the abundance of bacterial genera associated with water treatment were observed after rice cultivation in water. This difference in abundance may be due to the optimization of water conditions resulting from the presence of rice, which reduced the growth of those water treatment bacteria.

The functional analysis of the microbial community revealed that the water samples in the PRFB group showed an increased abundance of metabolic pathways related to nitrogen metabolism. Previous studies have shown that microbial nitrogen fixation is a crucial natural source of reactive nitrogen in floating treatment wetlands [67]. Additionally, in a combined treatment system comprising hydroponic plants and biofilm, nitrate reduction was identified as one of the main functions of the microbiota [68]. Therefore, it can be inferred that PRFBs enhance the microbiota’s function in water, specifically in nitrate reduction and nitrogen fixation, leading to an improvement in nitrogen metabolism in pond water, which was consistent with those previous results.

## 5. Conclusions

This study is the first report on the impact of rice cultivation on both water quality and the microbial community structure in ponds of mandarin fish fed an artificial diet. The results revealed that rice cultivation reduces nutrient salt levels in the water, regulating the aquatic environment. Simultaneously, rice cultivation modified the microbial community in the water by decreasing diversity and affecting its composition, particularly affecting bacteria that are involved in carbon, nitrogen, and phosphorus metabolism such as KD4-96, *Rhizorhapis*, *Dinghuibacter*, *Candidatus*_Aquiluna, *Chryseomicrobium*, and JG30_KF_CM45, resulting in their decreased abundance. Additionally, this study identified Proteobacteria, Actinobacteria, Firmicutes, Bacteroidetes, and Deinococcus-Thermus as the dominant bacterial phyla in ponds of mandarin fish fed an artificial diet. Furthermore, this research observed a distinct preference for the proliferation of Proteobacteria in the aquatic environment with rice cultivation on the water surface. These findings provided a theoretical foundation for the application of aquaculture of mandarin fish fed an artificial diet and rice floating beds.

## Figures and Tables

**Table 1:** Pond information.

Groups	Pond Number	Pond Area (hm^2^)	Density (Tail/hm^2^)	Rice Planting Area
Control	Control-A	0.234	60,000	0
	Control-B	0.330	60,000	0
	Control-C	0.310	60,000	0
PRFB	PRFB-A	0.240	60,000	30%
	PRFB-B	0.415	60,000	30%
	PRFB-C	0.330	60,000	30%

**Table 2:** Water quality parameters of the reservoir.

Water Quality Parameters	Alk	NH_4_^+^-N	NO_2_^−^-N	NO_3_^−^-N	TN	TP
Concentration (mg/L)	107.99	0.365	0.0225	0.024	1.508	0.425

**Table 3:** Water quality measurement methods.

Water Quality Parameters	Test Method
Alkalinity	pH indicator titration
NH_4_^+^-N	Nessler’s reagent colorimetric method
NO_2_^−^-N	N-(1-naphthyl) ethylenediamine photometric method
NO_3_^−^-N	Phenol sulfonic acid spectrophotometry
TN	Potassium persulfate ultraviolet spectrophotometry
TP	Malachite green phosphomolybdate heteropoly metalate spectrophotometry

## Data Availability

The sequencing data have been uploaded to the NCBI Sequence Read Archive repository at https://www.ncbi.nlm.nih.gov (accessed on 1 February 2023), reference number PRJNA1071861.

## Supplementary Materials

The following supporting information can be downloaded at https://www.mdpi.com/article/10.3390/biology13070549/s1, Table S1: Ingredients and chemical compositions of artificial feed; Table S2: Water quality in individual pond of different sampling periods.
