# Supplementary material for: Effect of Pond-Based Rice Floating Bed on the Microbial Community Structure and Quality of Water in Pond of Mandarin Fish Fed Using Artificial Diet
Source: Biology (Basel). 2024 Jul 21;13(7):549. doi: 10.3390/biology13070549 (PMC11274348; doi:10.3390/biology13070549)
Supplement: Supplementary file 1 [file biology-13-00549-s001.zip › biology-3092951-supplementary.pdf]

Supplemental Table 1. Ingredients and chemical compositions of artificial feed.

Ingredient	Proportion/%
White fish meal	40.00
Antarctic krill meal	10.00
Soy protein concentrate	8.00
Wheat gluten meal	5.00
Soybean meal	8.20
Spray-dried animal blood cells	4.00
High gluten flour	12.00
Squid paste	2.00
Yeast extract paste	2.00
Fish oil	2.00
Soybean oil	2.00
Soybean phospholipids	2.00
Calcium dihydrogen phosphate	1.00
Vitamin premix[1]	0.20
Mineral premix[2]	0.80
Choline chloride	0.50
Taurine	0.30
Total	100.00
Nutrient level[3]	
Moisture	8.51
Crude protein	52.62
Crude lipid	12.51
Crude ash	13.35
Starch	8.17

1 The vitamin premix provides the following per kg of diet: vitamin B1 20 mg, riboflavin 20 mg, vitamin B6 12 mg, vitamin B12 0.15 mg, vitamin K3 12 mg, inositol 300 mg, pantothenic acid 60 mg, niacin acid 70 mg, folic acid 10 mg, biotin 1 mg, vitamin A 8000 IU, vitamin D3 2000 IU, vitamin E 100 mg, vitamin C 500 mg, ethoxyquin 200 mg, defatted rice bran 535 mg; **2** The mineral premix provides the following per kg of diet: KCl 250 mg, KI (1%) 80 mg, $CoCl_2 \cdot 6H_2O$ (1%) 70 mg, $CuSO_4 \cdot 5H_2O$ 40 mg, $FeSO_4 \cdot H_2O$ 500 mg, $ZnSO_4 \cdot H_2O$ 500 mg, $MnSO_4 \cdot H_2O$ 200 mg, $Na_2SeO_3 \cdot 5H_2O$ (1%) 70 mg, $MgSO_4 \cdot H_2O$ 2500 mg, zeolite power 3790 mg; **3** Nutrient levels are measured values.

Supplemental Table 2. Water quality in individual pond of different sampling periods.

Control I	ALK	NH4+-N	TN	NO2--N	NO3--N	TP
Control-A	102.65±0.87	0.74±0.42	1.76±0.47	0.10±0.05	0.10±0.03	0.13±0.04
Control-B	111.71±2.64	1.83±0.16	3.20±0.55	0.14±0.08	0.04±0.02	0.27±0.09
Control-C	110.40±4.45	1.23±0.50	2.46±0.87	0.09±0.05	0.05±0.01	0.26±0.03
Control II						
Control-A	102.97±3.22	1.00±0.42	2.43±0.17	0.11±0.04	0.40±0.01	0.30±0.01
Control-B	105.22±1.53	2.14±0.27	4.47±0.76	0.30±0.05	0.50±0.05	0.35±0.04
Control-C	102.60±2.58	1.21±0.37	3.47±0.62	0.23±0.07	0.48±0.03	0.31±0.03
Control III						
Control-A	108.76±3.97	0.64±0.13	2.75±0.34	0.08±0.02	0.35±0.03	0.23±0.03
Control-B	119.37±1.71	0.44±0.18	3.56±0.55	0.25±0.08	0.32±0.06	0.27±0.07
Control-C	109.51±4.13	0.48±0.18	3.82±0.26	0.10±0.04	0.36±0.03	0.23±0.03
PRFB I						
PRFB-A	82.56±6.26	0.26±0.09	1.28±0.26	0.06±0.03	0.05±0.03	0.09±0.03
PRFB-B	93.98±3.18	0.31±0.03	1.26±0.28	0.06±0.04	0.09±0.04	0.09±0.04
PRFB-C	98.44±3.03	0.56±0.12	1.19±0.31	0.20±0.06	0.09±0.01	0.11±0.05
PRFB II						
PRFB-A	95.53±2.02	0.62±0.21	2.67±0.29	0.23±0.09	0.22±0.09	0.20±0.03
PRFB-B	95.65±0.67	0.20±0.04	1.56±0.30	0.09±0.04	0.45±0.01	0.13±0.03
PRFB-C	99.97±2.93	0.35±0.26	2.04±0.37	0.13±0.06	0.36±0.01	0.17±0.02
PRFB III						
PRFB-A	107.98±1.96 a	0.39±0.05	2.60±0.13	0.14±0.06	0.32±0.05 ab	0.27±0.04
PRFB-B	91.68±3.94 b	0.31±0.10	2.10±0.17	0.02±0.00	0.38±0.04 a	0.14±0.02
PRFB-C	111.91±3.40 a	0.35±0.09	2.33±0.12	0.04±0.00	0.20±0.02 b	0.18±0.02

Different letters indicate significant differences between different ponds of the same indicator in the same sampling period ($p < 0.05$).
